# A Metagenomic Survey of Wood Decay Fungi in the Urban Trees of Singapore

**DOI:** 10.3390/jof9040460

**Published:** 2023-04-10

**Authors:** Yan Hong, Jhing Yein Tan, Huiyu Xue, Mei Lun Chow, Mohamed Ali, Arthur Ng, Abigail Leong, Jeb Yeo, Shao Ming Koh, Megan Shi Ying Tang, Yan Yi Lee, Amy Mei Fun Choong, Serena Mei Lyn Lee, Riccardo Delli Ponti, Perry M. Chan, Daryl Lee, Jia Yih Wong, Marek Mutwil, Yok King Fong

**Affiliations:** 1 School of Biological Sciences, Nanyang Technological University, 60 Nanyang Drive, Singapore 637551, Singapore; jhingyein.tan@ntu.edu.sg (J.Y.T.); riccardo_ponti@bii.a-star.edu.sg (R.D.P.); mutwil@ntu.edu.sg (M.M.); 2 National Parks Board, 1 Cluny Road, Singapore Botanic Gardens, Singapore 259569, Singaporearthur_ng@nparks.gov.sg (A.N.); abigail_leong@nparks.gov.sg (A.L.); jeb_yeo@nparks.gov.sg (J.Y.); daryl_lee@nparks.gov.sg (D.L.); wong_jia_yih@nparks.gov.sg (J.Y.W.);; 3 Department of Biological Sciences, Faculty of Science, National University of Singapore, 14, Science Drive 4, Singapore 117543, Singapore; dbscmfa@nus.edu.sg; 4 Singapore Botanic Gardens, Singapore 259569, Singapore; serena_lee@nparks.gov.sg; 5 School of Applied Science, Nanyang Polytechnic, 180 Ang Mo Kio Avenue 8, Singapore 569830, Singapore; perry_chan@nyp.edu.sg

**Keywords:** wood rotting fungi, metagenomics, in vitro wood decay study, Rain Tree, Angsana, Yellow Flame, *Polyporales*, *Fulvifomes*, *Phellinus noxius*, *Ganoderma*

## Abstract

Mature tropical urban trees are susceptible to root and trunk rot caused by pathogenic fungi. A metagenomic survey of such fungi was carried out on 210 soil and tissue samples collected from 134 trees of 14 common species in Singapore. Furthermore, 121 fruiting bodies were collected and barcoded. Out of the 22,067 OTUs (operational taxonomic units) identified, 10,646 OTUs had annotation information, and most were either ascomycetes (63.4%) or basidiomycetes (22.5%). Based on their detection in the diseased tissues and surrounding soils and/or the presence of fruiting bodies, fourteen basidiomycetes (nine *Polyporales*, four *Hymenochaetales*, one *Boletales*) and three ascomycetes (three species of *Scytalidium*) were strongly associated with the diseased trees. *Fulvifomes siamensis* affected the largest number of tree species surveyed. The association of three fungi was further supported by in vitro wood decay studies. Genetic heterogeneity was common in the diseased tissues and fruiting bodies (*Ganoderma* species especially). This survey identified the common pathogenic fungi of tropical urban trees and laid the foundation for early diagnosis and targeted mitigation efforts. It also illustrated the complexity of fungal ecology and pathogenicity.

## 1. Introduction

Singapore’s green efforts started in the 1960s when the country was undergoing rapid industrialization and urbanization. Through careful planning and diligent execution, Singapore had become a world renowned “garden city” by the late 1980s. Even more trees were planted after that. By 2018, Singapore had reached a country-wide vegetation coverage of 49%, 13% more than in 1986 [1,2]. Initially, hardy and fast-growing species, such as Angsana (*Pterocarpus indicus*), Rain Tree (*Samanea saman*), Sea Apple (*Syzygium grande*), Broad Leaf Mahogany (*Swietenia macrophylla*), African Mahogany (*Khaya grandifoliola*) and Senegal Mahogany (*Khaya senegalensis*) were chosen. After that, more flowering trees such as the Yellow Flame (*Peltophorum pterocarpum*) and Trumpet tree (*Tabebuia rosea*) were planted, and fruit trees, such as the Sentul tree (*Sandoricum koetjape*) were planted. These same tree species were commonly planted across Southeast Asia and in other tropical/subtropical cities. These urban trees made significant contributions to keeping Singapore as one of the cites with the highest density of greenery in the world [3]. The trees provide a multitude of benefits including shade, temperature reduction, noise mitigation and pollution control. While beautifying the living environment, they are also a key component to Singapore’s climate mitigation plan, as such trees in Singapore store 4.1 million tons of carbon and can sequester around 17,500 more tons of carbon per year. This amount of stored carbon is equivalent to almost five times the total yearly household carbon emissions in the country [4]. Singapore aims to plant another one million urban trees by 2030.

Root and trunk rots are very damaging as they can lead to tree failure and the death of the trees. These diseases are also becoming a problem for the sustainable management of urban trees, and the detection of incipient decay would allow the early selection of the best mitigating actions. Generally, such diseases are caused by ascomycetes or basidiomycetes and their identification and characterization would lead to better tree management. However, most research on such problems has concentrated on temperate forest tree diseases, with the fungal diseases of tropical tree plantations being less well studied, and those of tropical urban parks and roadside trees even less. Therefore, there is a critical need for additional research on pathogenic fungi for tropical urban trees to allow for early detection and targeted mitigation.

Traditional methods of fungal identification generally rely on the macro- and micro- morphological characterization of their fruiting bodies, but this may be confounded by the effects of environmental plasticity [5]. DNA-based technology, such as DNA barcoding, has been increasingly used to assist in fungal identification. The barcoding technique uses a short, standardized DNA sequence called a “barcode” that is unique to each species. Utilizing primer sets that are generally applicable to the broadest possible taxonomic groups, PCR amplicons thus generated from the respective DNA isolations are sequenced and compared with a reference database for species identification. For animals and plants, this barcode usually targets a mitochondrial or chloroplast gene that evolves rapidly enough to provide sufficient molecular variation for species differentiation. In fungi, the barcoding region most commonly used consists of the region including the Internal Transcribed Spacer one (ITS1), the 5.8S ribosomal RNA gene and the Internal Transcribed Spacer two (ITS2), located between the 18S ribosomal RNA gene and the 28S ribosomal RNA gene [6]. While barcoding has become a widely used tool for the rapid and accurate identification of fungal species in research such as fungal ecology, systematics and biotechnology, traditional Sanger sequencing requires high-quality DNA and is only suitable for homogenous samples. In contrast, high-throughput next generation sequencing (NGS) protocols, with the construction of amplicon libraries and the subsequent massively parallel sequencing of the respective amplicons, allow for environmental samples with mixed fungal and microbial communities to be barcoded and identified, and for the relative abundances of the microorganisms to be determined.

This metagenomics approach, with the isolation and analysis of the total DNA recovered from a sample, allows scientists to study microbial communities without the need to isolate individual species, making it possible to study the full spectrum of biodiversity in a sample. This approach provides a more comprehensive view of microbial diversity and can reveal the functional roles that microorganisms play in their environment. Applications of metagenomics include identifying new microorganisms and understanding the interactions between microorganisms and their environment, as well as studying the microbiome’s role in human health and diseases. While most metagenomic studies of microbial communities have been conducted on bacteria, there are some successes in using this metagenomic approach to understand fungi diversity. Using DNA metagenomics data from hundreds of globally distributed soil samples, more than 70,000 operational taxonomic units (OTUs) were identified with 98% as the clustering threshold ratio. This study also found that fungal richness is decoupled from plant diversity [7]. A metagenomic study on fungal diversity in air samples collected from Seoul, South Korea identified three fungal phyla as the main contributors to fungal spores in air [8]. A recent metagenomic study surveyed the archeae, bacteria and fungi present on the Antarctic continent and the surrounding area [9]. There is, however, a noticeable lack of metagenomic application in the field of fungal phytopathology.

In order to understand wood/root rot fungi in Singapore’s urban trees, collaborative research was initiated with the National Parks Board of Singapore (NParks) to identify them in the diseased trees of the most common urban tree species. Soil samples around each tree, fruiting bodies and the diseased tissues behind fruiting bodies were collected. Soil samples were also obtained from different locations such as the primary forest and around adjacent healthy trees. In addition, healthy wood tissue samples were collected and analyzed as a reference. For heavily diseased trees that were eventually cut down and removed, more diseased tissues were collected from stumps and cut branches. Fruiting bodies were barcoded for molecular identities while a metagenomic analysis was performed on soil and diseased tissue samples. With >240 metagenomic datasets and fruiting body molecular identities, the potential threats were short-listed by checking their significant presence in diseased tissues with the concomitant presence of the same fungi in soil and/or fruiting bodies. The literature on the same or closely related fungi by other researchers was also taken into consideration. As a result, 17 wood decay fungal species of varying severity were identified for Singapore urban trees. Pure isolates of three fungal species (*Fulvifomes siamensis*, *Rigidoporus microporus* and *Phellinus noxius*) were used for in vitro wood decay studies as further validation.

## 2. Materials and Methods

### 2.1. Sample Collection

NParks staff carry out regular tree inspections on urban trees in Singapore. Those displaying symptoms of stunted growth or abnormal defoliation are further checked for the presence of fruiting bodies around the base of the tree or on the tree trunk. For suspected diseased trees, wood integrity is further examined by methods such as resistance drilling, or from visual cues. For this study, fruiting body samples were collected with all details (location, date, tree species, tree morphology, photograph with size references) recorded, and cambial or sapwood samples were collected for a fungal assessment where decay was observed.

Soil samples (each of ~40 g) around a selected tree were collected at ~10 cm below the surface, with the associated debris and/or weeds discarded. For trees with serious wood decay symptoms, multiple soil samples were collected from different locations around the tree. For other trees, samples from 3 locations around the tree were pooled as one sample. Some soil samples were also collected from adjacent healthy-looking trees to assess the presence of pathogenic fungi.

During or after diseased tree removal, more samples were collected from stumps and cut trunks for decayed wood tissue, with healthy wood pieces collected to serve as a reference.

### 2.2. Fungal Culture

Diseased tissues and fruiting bodies were disinfected with 70% ethanol before cutting open with a disinfected hand saw. The exposed interior was cut into small fragments. The fragments were placed equally spaced apart onto potato dextrose agar (PDA) or rose bengal agar (RBA) plates with antibiotics (streptomycin 30 mg/L + ampicillin 100 mg/L). Fungal colonies growing from the fragments were considered as the 1st generation (G1) isolates. Colonies were sub-cultured onto PDA or RBA plates with antibiotics to obtain pure cultures. Sub-culturing was performed by transferring hyphae from the edge of the fungal colony to a new culture plate with sterile scalpel blades. All plates were incubated at 30 °C in the dark to promote fungal growth.

### 2.3. DNA Isolation

Pooled soil samples were sieved and stored at −80 °C. Aliquots of 100 mg were subjected to total DNA isolation with the Qiagen PowerLyzer PowerSoil DNA Isolation kit (Qiagen) by following the manufacturer’s instructions. For fungal isolates, the lyticase-chelex 100 DNA isolation protocol for fungal mycelia [10] was used. Fruiting bodies and tissue samples were cut open with a sterile hand saw before small fragments were cut from the freshly exposed interior. The fragments were further grounded using a mortar with pestle in the presence of liquid nitrogen, and 100 mg of powered fragments was used for total DNA isolation by the same Qiagen PowerLyzer PowerSoil DNA Isolation kit.

### 2.4. Metagenomic Survey of Samples

The ITS1 gene was amplified with the Phusion^®^ High-Fidelity PCR Master Mix (New England Biolabs) with the primer pairs of 1737F (5′-GGAAGTAAAAGTCGTAACAAGG-3′) and 2043R (5′-GCTGCGTTCTTCATCGATGC-3′). PCR products were checked with electrophoresis on 2% agarose gel. Samples with a bright main band between 300 bp and 450 bp were chosen for further experiments. The mixed PCR products were purified with the Qiagen Gel Extraction Kit (Qiagen, Hilden, Germany). The libraries were generated with the NEBNext^®^ UltraTM DNA Library Prep Kit for Illumina Sequencing and Data Processing (NovogeneAIT Genomics, Singapore). Paired-end reads were merged using FLASH (V1.2.7) to obtain the raw tags, and high quality clean tags were selected using fastp software. Finally, Vsearch software was used to blast clean tags to the database to detect and remove chimeras, producing the final effective tags. The DADA2 or deblur module in the QIIME2 software was used to create the denoise (DADA2 were used by default) to obtain the final ASVs (amplicon sequence variables) and feature table. Then, the Classify-sklearn moduler in QIIME2 software was used to compare ASVs with the database (Fungi Unite database, https://unite.ut.ee, accessed on 31 January 2023 to obtain the species annotation of each ASV. Clustering was conducted through Spyder Python 3.9 (https://www.spyder-ide.org/, (accessed on 31 January 2023)) to implement sumaclust version 1.0.36 (https://git.metabarcoding.org/obitools/sumaclust/-/wikis/home, accessed on 31 January 2023) with 90.0, 95.0, 96.0, 97.0, 98.0 and 99.0% sequence similarity thresholds, respectively. A representative sequence of each cluster was determined to be the sequence with the highest sum of all pairwise similarity scores. These representative sequences were labelled as the main operational taxonomic units (Main OTU).

An Excel-based database of the Main OTUs was generated and had included the following details of every ASV sequence within each main OTU: sample name and type, host tree species, metagenomic absolute hit values, health status of the tree and third-party taxonomic annotations as far as identifiable against the UNITE (unite.ut.ee) database. Each Main OTU was also assigned a group ID for easy reference. For the statistical analysis of the fungi diversity distribution, each Main OTU taxonomic annotation included every unique taxonomic annotation(s) within the cluster. To verify the third party taxonomic annotations, BLASTn searches of selected ASV sequences against the NCBI database were conducted (http://ncbi.github.io/blast-cloud/dev/api.html, accessed on 31 January 2023) and implemented in Bio.Blast.NCBIWWW module Ver 1.75 (https://biopython.org/docs/1.75/api/Bio.Blast.NCBIWWW.html#module-Bio.Blast.NCBIWWW, accessed on 31 January 2023) (hitlist_size = 3, alignments = 30, descriptions = 30) through the Spyder Python 3.9 interpreter. Fungi names were checked against the Index Fungorum (http://indexfungorum.org/Names/Names.asp, accessed on 31 January 2023) for the current most-accepted names.

### 2.5. Barcoding for Fruiting Bodies and Fungal Culture

DNA from the fruiting body and fungal culture were PCR-amplified for ITS1-5.8S-ITS2 region with the V9D and LS266 primer pair (5′-TTAAGTCCCTGCCCTTTGTA-3′ and 5′-GCATTCCCAAACAACTCGACTC-3′, respectively) with the following program [10]: an initial denaturation at 95 °C for 5 min; followed by 35 cycles of 30 s denaturation at 95 °C, 30 s annealing at 50 °C and 30 s extension at 72 °C; and a final extension of 72 °C for 5 min. PCR amplicons were purified with the QiaQuick PCR purification kit (Qiagen) prior to submission for sequencing (Bio Basic Asia Pacific Pte Ltd., Singapore) with the primers ITS5 and ITS2 (5′-GGAAGTAAAAGTCGTAACAAGG-3′ and 5′-GCTGCGTTCTTCATCGATGC-3′, respectively). Sequence chromatograms were visualized for verification/manual correction, and consensus sequences were assembled by Geneious Prime (2022.0.1). Molecular identification was conducted through the NCBI-BLASTn search against the “Nucleotide collection (nr/nt) database” for non-redundant GenBank entries. The molecular identity for the fruiting body was taken as the best match according to the E value (less than the cut-off value of 1-E100) with full annotation down to the species level.

### 2.6. In Vitro Wood Decay Study with Autoclaved Wood Blocks

The in vitro wood decay study was conducted according to Burcham [11]. Briefly, tree branches were collected during the regular pruning of healthy trees (>10 years old). Tree branches of 8–10 cm diameters were processed into sap wood blocks of 30 × 10 × 8 mm, which were then double autoclaved, and dried in the oven at 100 °C for 48 h to reach a constant weight. The dry weight of all the wood blocks was measured. Two wood blocks were placed onto one PDA Petri dish of 90 mm diameter that had been fully colonized by the test fungus. The assembly was sealed and incubated at 28 °C for 12 weeks. A total of 20 wood blocks were used for each inoculation experiment and the reference group (CK) had the same number of wood blocks treated the same way in uninoculated PDA Petri dish. Twelve weeks later, all the fungal growth was scraped from the wood blocks, which were subsequently wiped clean with tissue paper before drying in the oven at 100 °C for 48 h. The dry weight of all wood blocks was measured. The dry weight for each block after the treatment was computed as a percentage of its dry weight before the treatment. The average values and standard deviation (SD) values for each group (n = 20) were calculated using the statistical functions of MS Excel. Student’s *t*-test was conducted between an inoculation group and its reference group to check for statistical significance in weight loss after incubation with a test fungus.

## 3. Results

### 3.1. Overview of Surveyed Samples

A total of 347 samples from 134 trees belonging to fourteen species were taken by our survey (Table 1). Detailed sampling information can be found in Supplementary Table S1.

For those diseased trees that were subsequently removed, multiple soil, fruiting body and diseased tissue samples were taken prior to and after tree removal. The five tree species with more than 10 individuals sampled were the Rain Tree, Angsana, Khaya, Casuarina and Yellow Flame. Also included were some fungal fruiting bodies collected from unidentified tree logs, and three soil samples from a primary forest area to serve as reference points.

Each sample ID starts with a tree code, followed by the serial number for the tree and ends with the sample type, e.g., R16FB is a fruiting body sample collected from a Rain Tree with serial number 16.

### 3.2. Barcoding for Fruiting Bodies

Most of the fruiting bodies could be directly barcoded, producing high quality sequences with distinctive chromatogram peaks, indicating the presence of a single genotype within a fruiting body. This was the case for most *Fulvifomes siamensis* fruiting bodies and *Rigidoporus microporus* fruiting bodies. A number of fruiting body samples gave poor quality sequences, with only restricted regions of the chromatograms having high quality readings, or regions with multiple overlapping chromatogram peaks. This was the case for most *Ganoderma* fruiting bodies and all *Phellinus* fruiting bodies. Samples that failed to give good sequencing were sent out for a metagenomic analysis to assess the possibility of genetic heterogeneity and their relative abundances when they existed.

### 3.3. Metagenomic Analysis Reveals a Rich Funga Diversity

Full metagenomic data for 245 samples were obtained, each with 50,000–100,000 clean sequence reads, which were further assigned to various genotypes in the form of final ASVs (amplicon sequence variables). Using 90.0, 95.0, 96.0, 97.0, 98.0 and 99.0% sequence similarity thresholds, clustering revealed 13,953, 18,146, 19,399, 21,154, 22,067 and 23,356 main OTUs (operational taxonomic units), respectively. Furthermore, 98% was used as the cut-off threshold for the final clustering to be consistent with a previous study [7] on global soil funga diversity. There were a total number of 22,067 main OTUs with a minimum of five sequence reads. An Excel database was built (available as the Supplementary Table S2) with the detailed information on the main OTUs and their composition ASVs with sample name and type, host tree species, metagenomic absolute hit values, health status of the tree and third-party taxonomic annotations.

It should be noted that more diversities may be present in the composition ASVs. Within a main OTU, composition ASVs can have single nucleotide polymorphisms (SNP) among them and may not share the same distribution pattern.

Among the total number of 22,067 OTUs, 10,775 of them had annotation information (Table 2). Most belong to Phylum *Basidiomycota* and *Ascomycota* (2426/22.5% and 6826/63.3%, respectively). Class *Sordariomycetes* was the largest *Ascomycota* class (2556/37.4%) and *Agaricomycetes* was the dominant *Basidiomycota* class, accounting for 81.7% of the total (1983/2426). *Agaricales* and *Polyporales* were the most abundant orders under class *Agaricomycetes*. The order *Polyporales* including many wood decay fungi was well represented with 237 OTUs.

After combining fruiting body barcoding and metagenomic analysis results, the identification was performed for the fungal species with high relative OTU abundance in the diseased tissues (>10% relative abundance), and concomitantly present as fruiting bodies and/or in soil samples for the same tree. The literature on their roles in wood decay was also taken into consideration. The following wood decay fungi were shortlisted (Table 3). These pathogens were not present in the reference soil samples collected from the primary forest or in healthy tissue/wood samples.

Tree species are represented by tree codes (see Table 1). The number of trees infected is indicated within brackets after tree codes, and the total sample number is also given if it is more than the tree number. The cut-off value for metagenomic sequence reads is 500 for soil samples, >10% relative abundance in fruiting bodies and/or diseased tissues.

### 3.4. Wood Decay Fungi from Singapore Urban Trees

#### 3.4.1. *Rigidoporus microporus*

*R. microporus*, a well-known root-infecting fungal pathogen whose rhizomorphic structures can reach suitable hosts several meters away [2], is known to cause white rot root disease in more than 100 different tree species. It was first identified in 1904 as a pathogen of the rubber tree (*Hevea brasiliensis*) in the Singapore Botanic Gardens [12], and is regarded as the most economically important pathogen of the rubber tree.

**Figure 1:**
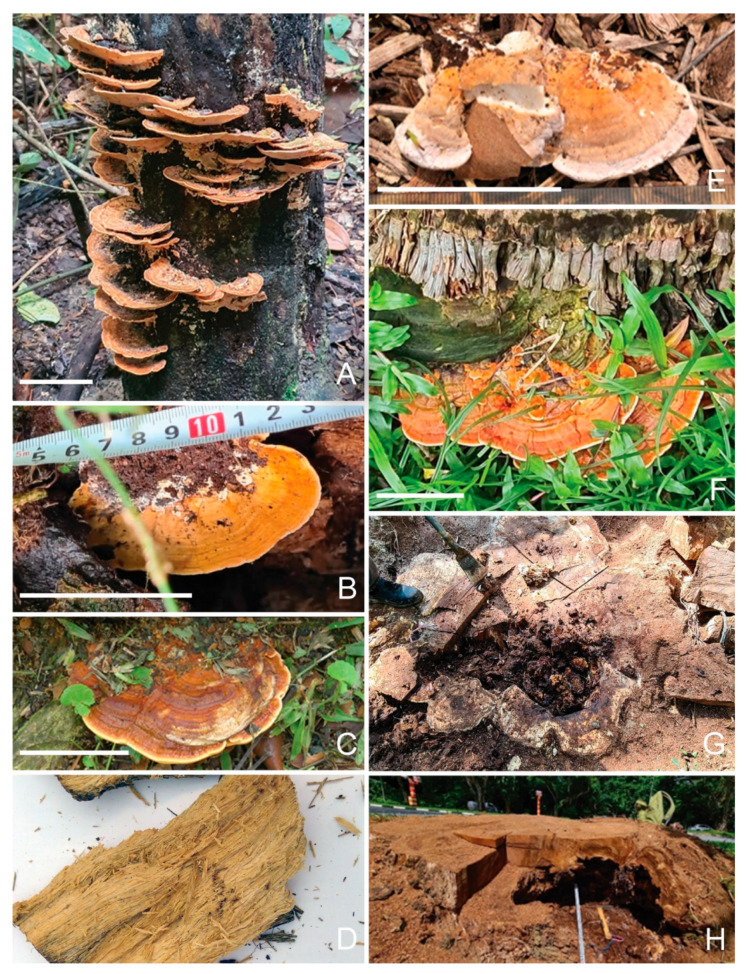
*Rigidoporus microporus* detected in Singapore urban trees. (**A**): a tree log with *R. microporus* fruiting bodies (L132); (**B**,**C**): a fruiting body at the base of a decayed Rain Tree (R16, R66); (**D**): a piece of decayed wood from a Rain Tree (R70) infected by *R. microporus*; (**E**): a similar-looking fruiting body of *Meripilus giganteus* (A164FB3); (**F**): a palm stump (P175) with *R. microporus* fruiting body; (**G**): a Rain Tree stump decaying in the heart (R33) by *R. microporus*; (**H**): a Rain Tree stump (R16) with a big cavity infected by *R. microporus*. Bar = 5 cm.

This study has identified a total of 13 OTUs of *R. microporus* (Table 3), which were widely present in soil samples collected from diseased Rain Trees, making up to 33% of a sample’s total reads (R48), and were similarly detected as fruiting bodies and in the diseased tissues of the respective Rain Trees (Figure 1B–D,G,H). Such a concomitant presence suggests possible pathogenicity for the Rain Tree. The rhizomorphic spread of *R. microporus* to neighboring Rain Trees may have also resulted in infected Rain Tree clusters, where *R. microporus* was detected in the soil samples of both the diseased tree and the neighboring non-symptomatic trees.

*R. microporus* was also detected in the diseased tissue samples and surrounding soil samples of a number of diseased Casuarina trees, as well as in Yellow Flame and Khaya trees. A Cabbage Palm stump was found with *R. microporus* fruiting bodies (Figure 1F, GenBank no. OQ558868, OQ558870), possibly of saprophytic growth.

Different *R. microporus* OTUs can co-exist in the same diseased samples. Diseased tissues for one Rain Tree (R33) had both OTU_3_Deca20 (Gp 32178) and OTU_5758_Deca20 (Gp32166) with relative abundances of 35% and 46% (for R33ITID).

#### 3.4.2. *Meripilus giganteus*

A *Rigidoporus microporus*-like fruiting body was collected from an Angsana tree base (Figure 1E, A164FB3) and later barcoded as *Meripilus giganteus* (GenBank no. OQ569361). It was also present in the diseased tissue inside the site of infection (A164DT3). *Meripilus giganteus* is another Polyporales fungus in the family Meripilaceae. It causes white rot in various broadleaved trees such as beech, and also *Pinus* and *Ulmus* species. Being a widely distributed heartwood rot fungus in Europe [13], this is the first identification in the tropics and association with Angsana wood decay.

#### 3.4.3. *Ganoderma* Species

*Ganoderma* is a genus of Polyporales fungi in the family of *Ganodermataceae*. *Ganoderma* species are characterized by double-walled basidiospores that are large, perennial and woody brackets. They are lignicolous and leathery either with or without a stem. *G. boninense* (=*G. orbiforme*), *G. zonatum* and *G. miniatocinctum* are reported to be responsible for basal stem rot disease in Asian oil palm plantations [14]. *G. australe* and *G. applanatum* are associated with widely planted acacias in East Asia and the Pacific region [15].

Two species of *Ganoderma* are shortlisted as threats to Singapore urban trees.

##### *Ganoderma* *orbiforme*

**Figure 2:**
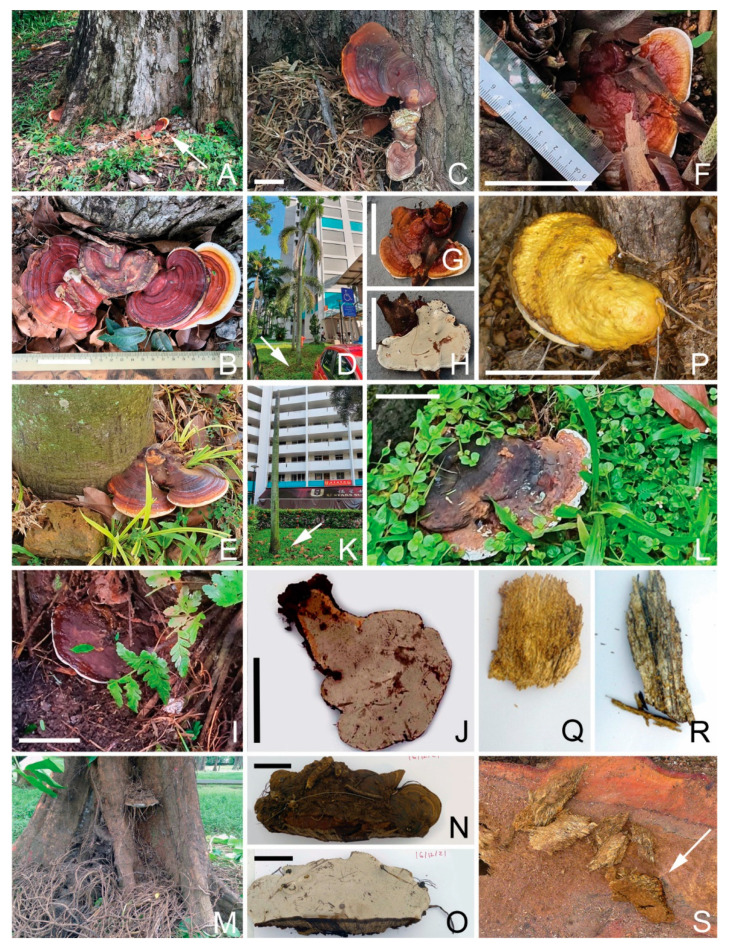
*Ganoderma species* detected in Singapore urban trees. (**A**,**B**): A *G. tropicum* fruiting body on an Angsana tree (A143); (**C**): a *G. tropicum* fruiting body on a Casuarina tree (CE41FB); (**D**,**E**): a *G. orbiforme* fruiting body on a Foxtail Palm (P168FB); (**F**–**H**): a *G. orbiforme* fruiting body on a Yellow Cane Palm (P172FB1); (**I**,**J**): a *G. australe* fruiting on a Yellow Cane Palm (P172FB2); (**K**,**L**): a bold head Foxtail Palm with *G. orbiforme* fruiting body at base (P174FB); (**M**–**O**): a big *G. australe* fruiting body from a Casuarina tree (CE153FB); (**P**): a *Tomophagus colossus* fruiting body on an Angsana tree (A126); (**Q**): a decayed wood by *G. australe* (CE149DT4); (**R**): a decayed wood from the same tree by *Fulvifomes siamensis* (CE149DT5); (**S**): a Casuarina stump (CE149) with a visible strip of decayed wood (arrowed) infected by *G. australe*. Bar = 5 cm.

The palm-specific phytopathogen *G. orbiforme* was detected in multiple fruiting bodies growing near the base of Foxtail Palms and Yellow Cane Palms collected from a single location (Figure 2D–L), and in soil samples collected from some Red Sealing Wax Palms in the Singapore Botanic Gardens of Singapore.

##### *Ganoderma* *australe*

It is a well-known white rot fungus. Its fruiting bodies were found on palms, Casuarina and Rain Tree. It was also present in diseased tissues in Casuarina and palms.

#### 3.4.4. *Tomophagus colossus*

*Tomophagus* is a *Basidiomycete* fungus genus in the family *Ganodermataceae*. It is characterized by the very light weight of the basidiocarp; the floccose; soft, spongy and light colored context; and friable tubes [16]. *Tomophagus colossus* fruiting bodies were collected from Angsana trees (Figure 2P, GenBank no. OQ558872), and were also a dominant presence in the diseased tissue collected from the same tree.

#### 3.4.5. *Fulvifomes siamensis*

In a study of stem/butt rot of *Xylocarpus granatum* trees at Hat Khanom-Mu Ko Thale Tai National Park, Thailand (rot incident of ~85%) [17], 92 basidiomes were collected and 46 fungal strains were sequenced in LSU and ITS regions. All of them belonged to a new *Fulvifomes* group that was phylotypically and phylogenetically distinctive from other known *Fulvifomes* species. The potential new species was tentatively named *Fulvifomes* sp. SP-2012b, and later named *Fulvifomes siamensis* [18].

From our metagenomic survey of soil and diseased samples collected in and proximal to urban trees, five strains were identified in Singapore with nearly identical sequences with the *Fulvifomes siamensis* reported. They were detected in almost all the urban trees surveyed. Multiple fruiting bodies were also identified by molecular barcoding to this species (GenBank no. OQ558844, OQ558845, OQ558846, OQ558847, OQ558848, OQ558849, OQ558850). Their OTUs were also consistently detected in soil, fruiting bodies and diseased tissues of the following urban tree species:

- Rain Tree;
- Casuarina;
- Khaya tree;
- Yellow Flame;
- Sea Apple;
- Broad Leaf Mahogany.

Comprehensively, this provides evidence for its potential broad pathogenicity to the above six important urban trees in Singapore. It was also detected in the diseased tissue of Angsana and Tembusu trees, and as a fruiting body on an Angsana tree. However, while the metagenomic analysis has identified the presence of *Fulvifomes siamensis* in the diseased tissue of Tembusu (CF77DT1), there was no significant presence in soil near this tree or other tree tissue samples collected nearby. In addition, *Fulvifomes siamensis* was detected in the surrounding soils of some ornamental palms, but never in the diseased tissue or as fruiting bodies.

**Figure 3:**
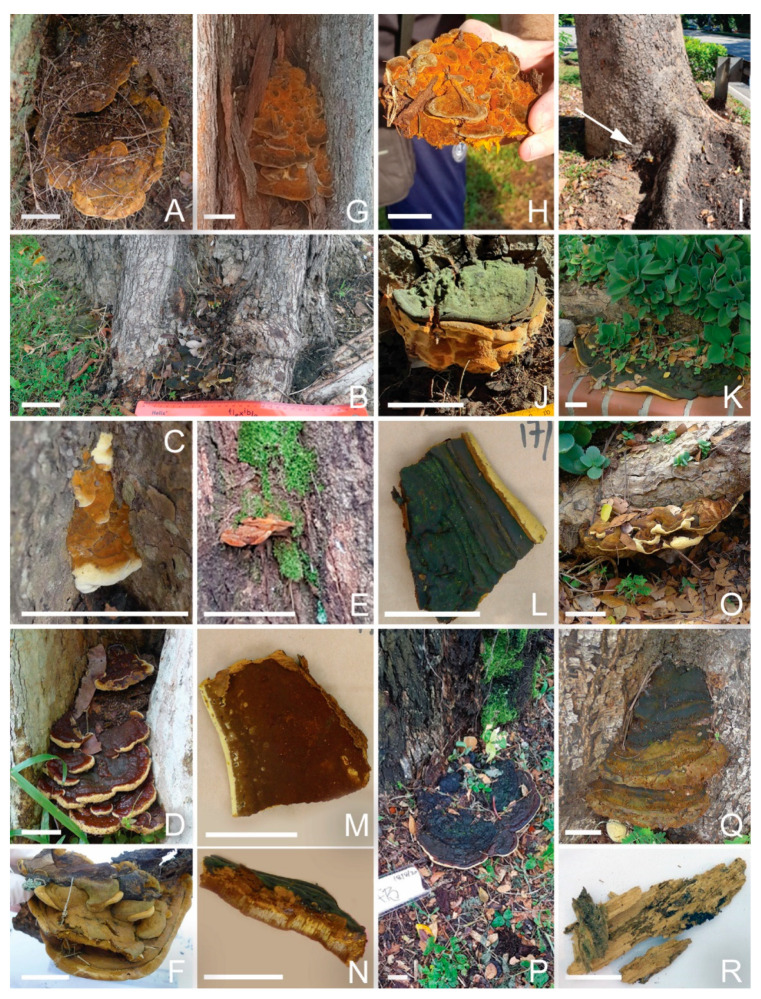
*Fulvifomes siamensis* detected in Singapore urban trees. (**A**): a fruiting body on a Casuarina tree (CE151); (**B**–**D**): fruiting bodies on *Syzygium grande* trees (SG62, SG63 and SG65, respectively); (**E**): a small fruiting body on Angsana tree trunk (A35); (**F**): a fruiting body from a *Swietenia macrophylla* tree (SM136); (**G**,**H**): a fruiting body on a Casuarina tree (CE39); (**I**,**J**): a fruiting body on a Khaya tree (KS138); (**K**): a large fruiting body on a Rain Tree (R67FB); (**L**–**N**): pileus surface, pore surface and cross-section of a piece of the fruiting body (R67FB); (**O**): another fruiting body on the same tree (R67); (**P**): a fruiting body on one Rain Tree (R32); (**Q**): a fruiting body on one Yellow Flame tree (YF68); (**R**): a piece of Angsana decayed wood infected by *Fulvifomes siamensis* (A64). Bar = 5 cm.

It should be noted that despite identical or highly similar ITS1 sequences, *Fulvifomes* fruiting bodies exhibited wide morphological diversity. Its upper face color ranges from bright orange (Figure 3H), to brown (Figure 3A,D) to black (Figure 3P). Some of them have distinctive white fringes (Figure 3C,D) while the white fringe is not obvious for others. Fruiting bodies can be in the form of clumps (Figure 3F,H,J) or as the multilayer growth (Figure 3D), and they can also have a flat fruiting body with sideways growth, as shown by the fan-like large fruiting body on a Rain Tree growing in a planting box (Figure 3K,L–N). All of these suggest morphological plasticity that makes the morphology-based diagnosis challenging.

As with *R. microporus*, *Fulvifomes siamensis* detection tends to happen in clusters. For the Rain Tree, multiple trees alongside one road were found with fruiting bodies and many had diseased tissues behind the fruiting bodies, suggesting ongoing pathogenesis. The presence of fungus in the soil samples suggests the soil acts as the pathogen reserve and source of new infections. The same situation happened for multiple Khaya trees. A third cluster was also found, where multiple Rain Tree and Yellow Flame trees were found with *Fulvifomes siamensis* fruiting bodies.

Its presence in soil can be significant, with as much as 32% (R25D2) of the total metagenomic sequence reads for the sample.

#### 3.4.6. *Phellinus noxius*

As a member of the order Hymenochaetales, this well-studied white rot fungus can infect more than 200 hardwood softwood trees. It is a major tree disease pathogen in Taiwan, causing the gradual to fast decline of the different trees [19]. It was found to be associated with the heart rot of *A. mangium* in Peninsular Malaysia and/or East Kalimantan, Indonesia [15].

**Figure 4:**
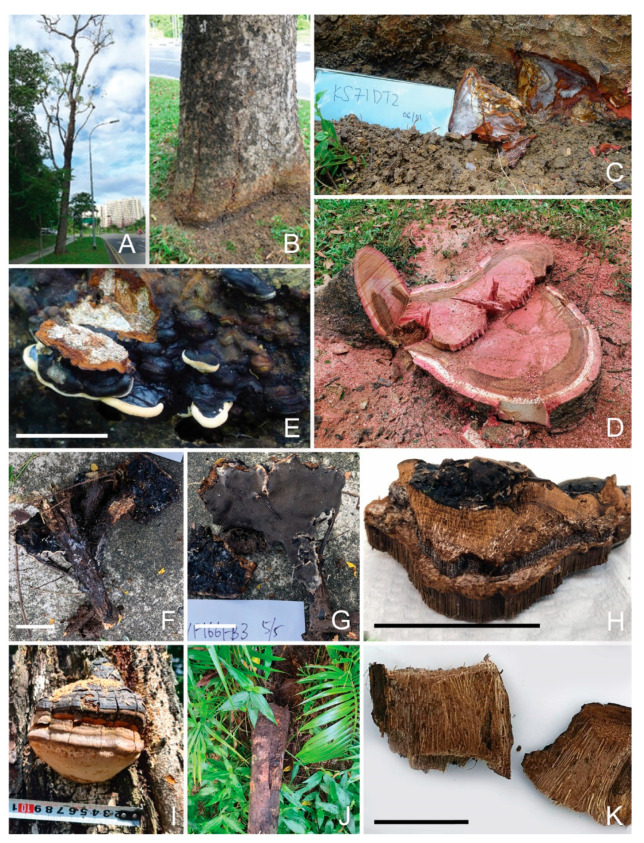
*Phellinus* species detected in Singapore urban trees. (**A**): A diseased Khaya tree (KS71); (**B**): a black ring near trunk base of the Khaya tree (KS71); (**C**): bark area (KS71DT2) with *P. noxius* mycelia; (**D**): KS71 tree stump with a ring of decayed wood infected by *P. noxius*; (**E**): a mosaic fruiting body of three *P. noxius* strains on a log of unknown species (L129); (**F**,**G**): a *P. noxius* fruiting body attached to a fallen branch near a Yellow Flame tree (YF166, pileus surface and pore surface); (**H**): cross-section of YF166FB3; (**I**): a *P. pachyphloeus* fruiting body on a Yellow Flame tree trunk (YF126FB); (**J**): a Yellow Cane Palm with basal decay (P171); (**K**): the Yellow Cane Palm stem, decayed by *P. noxius* and *Ganoderma* species (P171DT). Bar = 5 cm.

In this study, this fungus had a dominant presence in eighteen diseased tissues collected from nine Khaya trees. One example is a Khaya tree that was almost devoid of leaves (KS71, Figure 4A). There was a black stripe at soil level (Figure 4B) with white mycelia growth beneath the bark (Figure 4C). The pure fungal isolate was obtained from infected tissues beneath the bark (GenBank no. OQ558863). Excavation of the tree revealed a brownish circle of decayed sap and heart wood (Figure 4D), for which multiple samples were found dominated by *P. noxius*. There were no *P. noxius* fruiting bodies on any of the surveyed Khaya trees.

A dark-colored fruiting body was collected from a fallen branch near a Yellow Flame tree (YF166FB3) and was later identified as *Phellinus noxius*, consisting of two different OTUs.

Our research identified a big number of *P. noxius* OTUs (16) in Singapore. The presence of three different *P. noxius* OTUs was also found in one fruiting body collected from one unidentified log (L129, Figure 4E). These three distinctive OTUs (OTU_12_Oct21, OTU_179_Oct21 and OTU_458_Oct21) had relative abundances of 34%, 15% and 50%, respectively. This finding suggests that it was a mosaic fruiting body (inter-strain heterogeneity).

Two *P. noxius* OTUs were the dominant presence in a decayed yellow-cane palm stem (P171DT), accompanied by OTUs of two *Ganoderma* species.

It was also detected occasionally in both soil and diseased tissue samples of Rain Tree, Tembusu, Sea Apple and Casuarina, thus suggesting, at best, a less important pathogenic relationship.

#### 3.4.7. *Serpula similis*

**Figure 5:**
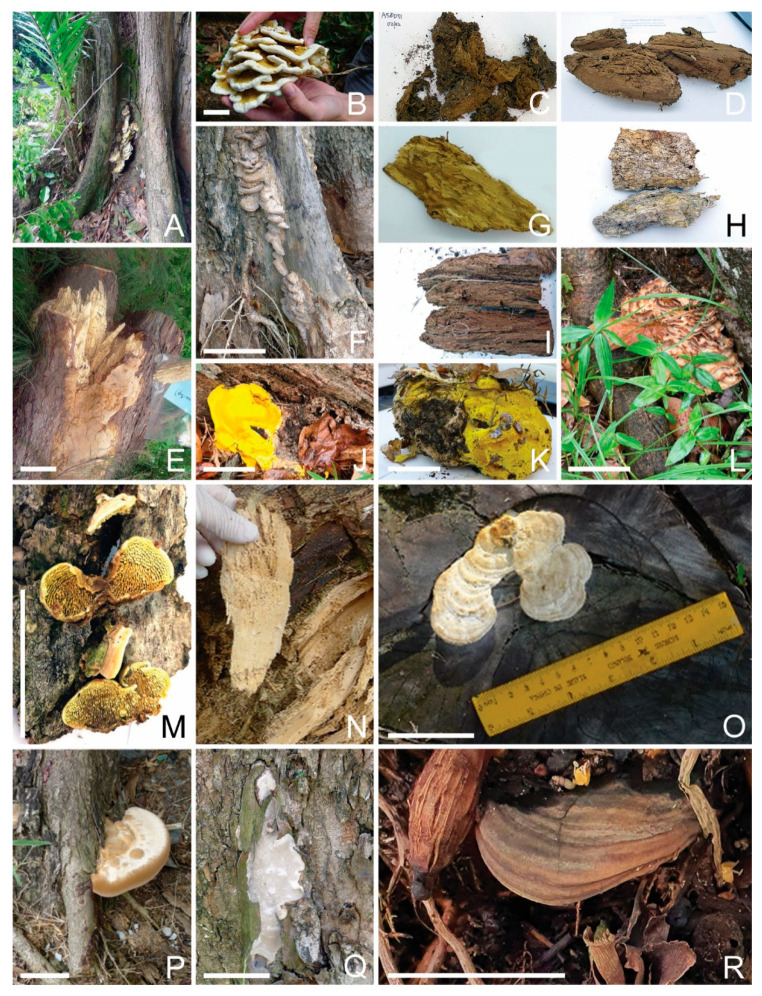
Other pathogenic fungi detected in Singapore urban trees. (**A**): An Angsana tree (A58) with *Serpula similis* fruiting bodies; (**B**): *Serpula similis* fruiting bodies (A58FB); (**C**): decayed wood from the same tree (A58DT1); (**D**): decayed Angsana wood dominated by *Serpula similis* from another tree (A34); (**E**): crust fungus *Fomitiporia torreyae* fruiting body on a Casuarina branch with decayed wood behind (CE151DT2); (**F**): *Fomes meliae* on an Angsana tree (A127FB); (**G**): a decayed piece of Angsana wood infected by *Fomes meliae*; (**H**): a decayed Yellow Flame wood infected by *Fomitiporia bannaensis* (YF37DT1); (**I**): a decayed Yellow Flame wood infected by *Gloiothele lactescens* (YF37ST1); (**J**): a *Pseudofibroporia citrinella* fruiting body on an Angsana tree (A36); (**K**): the spongy fruiting body of *Pseudofibroporia citrinella* (A36FB); (**L**): *Phlebia acanthocystis* fruiting bodies on a Purple Millettia tree (PM163); (**M**): *Flavodon flavus* fruiting bodies on an Angsana tree (A165); (**N**): an Angsana decayed wood piece infected by *Phlebiopsis ravenelii* (A128DT2); (**O**): a *Trametes gibbosa* fruiting body on a Khaya tree stump (KS122); (**P**): *Leiotrametes lactinea* fruiting bodies on an Angsana tree (A80); (**Q**): *Perenniporia tephropora* crust fruiting body on Angsana tree (A144); (**R**): *Scytalidium ganodermophthora*-infected *Ganoderma* basidiome at base of a Yellow Cane Palm (P171FB). Bar = 5 cm.

A member of the order Boletales, these brown rot fungi were found in the diseased tissues of an infected Angsana tree (A58, Figure 5A,B). Fruiting bodies were found on the lower stem of the same tree (GenBank no. OQ558871). In another Angsana (A34) tree, three distinct OTUs of this fungus were found on diseased tissues (Figure 5C,D). The diseased wood tissues were dry, crispy and brown in color, consistent with the expected brown rot phenotypes. The fungus was detected in the soil samples around this tree (A34D1, D2 and D3). It co-existed with other fungi in decayed wood, such as *Rasamsonia emersonii*, *Scytalidium* sp. and *Fulvifomes siamensis,* serving as one example for the involvement of multiple fungi in the decay of wood [20]. In addition, *S. similis* was detected in soil samples from a Rain Tree (R66D) and a diseased tissue sample from a Khaya tree (KS57D1).

#### 3.4.8. *Fomitiporia bannaensis*

*Fomitiporia* is a genus of the family *Hymenochaetaceae*. *Fomitiporia bannaensis* is rarely reported to be associated with wood decay in trees. This fungus was identified by our study to be present in the soil and as a fungus crust with white decayed wood behind a Casuarina tree branch (Figure 5E). Our study also found its presence in Yellow Flame and Khaya trees’ decayed wood, suggesting its wider pathogenic spectrum.

#### 3.4.9. *Fomes meliae*

*Fomes meliae* is a wood decay species of basidiomycetes fungus which causes brown rot mostly in softwood and in some species of hardwood. The same species has been explored for the high level of expression of endoglucanase production [21]. *Fomes meliae* is reported to be a plant pathogen that causes wood rot in nectarines, peaches [22] and *Platanus* sp. (Sycamore). A crust fruiting body collected from an Angsana tree (A127) was molecularly identified as *Fomes meliae* (Figure 5F, GenBank no. OQ558843). This fungus also had a significant presence in the soft light yellowish decayed wood sample from this tree and its neighbor (A128). As *Fomes meliae* has only been reported as a disease in temperate trees, its pathogenicity towards Angsana will have to be determined through Koch’s postulate experiments.

#### 3.4.10. *Perenniporia tephropora*

A white color crust-like fungus fruiting body was collected from one Angsana tree trunk (Figure 5Q, A144FB, GenBank no. OQ558861) and barcoded as *Perenniporia tephropora,* a white rot fungus. Diseased tissues were also collected behind this fruiting body and another location on the same tree trunk (A144). A metagenomic analysis identified *Perenniporia tephropora* as the dominant presence.

#### 3.4.11. *Pesudofibroporia citrinella*

One fruiting body of this *Polyporales* fungus was collected from the root zone of an Angsana tree (A36, Figure 5J,K, GenBank no. OQ558866). It was also found in diseased tissues collected from one Yellow Flame tree (YF37ST1 and YF37ST2).

#### 3.4.12. *Flavodon flavus*

Multiple fruiting bodies of this fungus were found at the trunk of an Angsana tree (A165FB, Figure 5M, GenBank no. OQ558842). It is a common wood decay fungus. A metagenomic analysis also revealed its presence in the diseased tissues for this tree and its neighboring Angsana tree (A164DT1/DT3, A165DT1/DT3). *Flavodon flavus* (as *Irpex flavus*) has been reported as a pathogen of Angsana in Malaysia [23].

#### 3.4.13. *Scytalidium* sp.

The ascomycota microfungi have been reported to participate in the final stage of wood decay. *Scytalidum lignicola* produces soft rot decay in Douglas fir [24]. Three *Scytalidium* species were detected in the soil and diseased wood tissue samples: *Scytalidium lignicola, Scytalidium circinatum* and *Scytalidium cuboideum*. All three were detected in Angsana, but only *Scytalidium lignicola* was detected in Casuarina, and in the diseased tissues of Purple Millettia.

### 3.5. Notes on Other Fungi Identified

#### 3.5.1. *Scytalidium ganodermophthora*

A unique-looking fruiting body was collected at the base of a Yellow Cane Palm (P171). It looked similar to a *Ganoderma* fruiting body but had brown/yellow coloration throughout (Figure 5R). A metagenomic analysis revealed it to be mostly *Scytalidium ganodermophthora* (57% relative abundance, GenBank no. OQ572648, OQ572649), an ascomycete that has been reported to be the most destructive fungus for *Ganoderma lucidum* farming in Korea [25]. Within the same clump of Yellow Cane Palm, *Ganoderma* fruiting bodies were found growing at the bases of other palms. *Phellinus noxius* and *Ganoderma* sp. were identified from a decayed stem within the same clump (Figure 4J,K). It is suggested that this *Ganoderma* pathogen had hyperparasitized the *Ganoderma* fruiting body. This fungus was also found in one diseased tissue of Casuarina, also suggesting possible hyperparasitism of the wood decay fungi present. In view of its parasite growth on the *Ganoderma* fruiting body, it holds some potential for the biocontrol of the *Ganoderma* species.

#### 3.5.2. *Gloiothele lactescens*

This fungus was present in soil samples around Casuarina trees and Rain Trees. It was also found in the diseased tissues for one Rain Tree (R45, GenBank no. OQ572604). It was the dominant presence in two decayed wood samples inside a Yellow Flame trunk (GenBank no. OQ572603). An unusual morphology for the decayed wood was the presence of a long and thick string of white mycelia (Figure 5I). Due to the low homology score (3e-39), its identity was not conclusive, and it was likely a new species.

#### 3.5.3. *Phellinus pachyphloeus*

A fruiting body with a thick crust and yellowish-brown punky context was collected from the trunk of a Yellow Flame tree (Figure 4I), and direct barcoding identified it as *Phellinus pachyphloeus* (GenBank no. OQ558855). This fungus has been reported on tree trunks in forest in the states of Johor, Negeri Sembilan, Pahang, Perlis and Selangor [26] of Malaysia. It is both parasitic and saprophytic, causing sap and heart rot in angiosperms, especially species of *Ficus* and *Mangifera*, and produces white stringy rot [27]. A gigantic fruiting body measuring 151 × 142 × 57 cm on a mango tree was reported in India [28]. This was also the first detection of this fungus in Singapore.

#### 3.5.4. *Phlebia acanthocystis*

A fruiting body (PM163FB) was collected from the root zone of a Purple Millettia tree (Figure 5L, GenBank no. OQ558865), but it had little presence in the tissue behind the fruiting body.

#### 3.5.5. *Perenniporia tenuis*

One fruiting body of the white rot fungus *Perenniporia tenuis* was found in another Angsana tree (A81, GenBank no. OQ558860), about 60 m away from A80. Its fruiting body was sessile.

#### 3.5.6. *Leiotrametes lactinea*

Two fruiting bodies of this white rot fungus were found at the root flare of an Angsana tree (A80, Figure 5P, GenBank no. OQ558859), with no stem and a half-circle bread appearance.

#### 3.5.7. *Trametes gibbosa*

A fruiting body growing on the stump of a Khaya tree was barcoded as *Trametes gibbosa* (Figure 5O, GenBank no. OQ558873), a white rot Polyporales commonly known as the lumpy bracket. It is often found on beech stumps and the dead wood of other hardwood species. It is likely saprophytic, and hence not a concern for living trees.

#### 3.5.8. *Vanderbylia fraxinea*

Its fruiting body was collected from the surface root of one Rain Tree (GenBank no. OQ558875) and was detected in the diseased tissue behind. It is probably pathogenic to the Rain Tree.

#### 3.5.9. *Ganoderma tropicum*

Fruiting bodies of this Ganoderma species were collected from an Angsana tree (Figure 2A,B, GenBank no. OQ572601, OQ572602). However, it had no significant presence in any diseased tissue, suggesting its limited impact on wood integrity.

### 3.6. Presence of Multiple Fungal Species in Diseased Trees

Our metagenomic survey uncovered the presence of multiple fungal species in the same diseased trees. Examples include:

- Yellow Flame tree (YF37): Three fruiting bodies of *Fulvifomes siamensis* were collected from this tree, but its presence in the decayed wood samples collected after tree removal was minor (<5%). Instead, *Fomitiporia bannaensis* (two strains, with relative abundance of 59% and 40% for DYF37T1) and *Gloiothele lactescens* (with relative abundance of 58% and 85% in DYF37T3 and DYF37DTb, respectively) were the dominant presence in the decayed wood samples.
- Casuarina: A decayed wood sample from the stump of a Casuarina (ES134) had both *Rigidoporus microporus* and *Ganoderma australe* (36% and 48% relative abundance, respectively).
- Rain Tree: *Rigidoporus microporus* and *Fulviformes siamensis* are generally mutually exclusive with each other in soil samples. Likewise, Rain Trees’ diseased tissues collected from trees in the same location were either dominated by *Rigidoporus microporus* (R28D1 and R32D1) or *Fulvifomes siamensis* (R25D2, R29D2, R31D2). In rare cases, they could also co-exist in diseased tissues, such as R33DTD, with two OTUs of *Rigidoporus microporus*, two OTUs of *Fulvifomes siamensis* and two unknown fungi (with relative abundance of 15%, 13%, 3%, 2%, 9% and 20% respectively).

**Figure 6:**
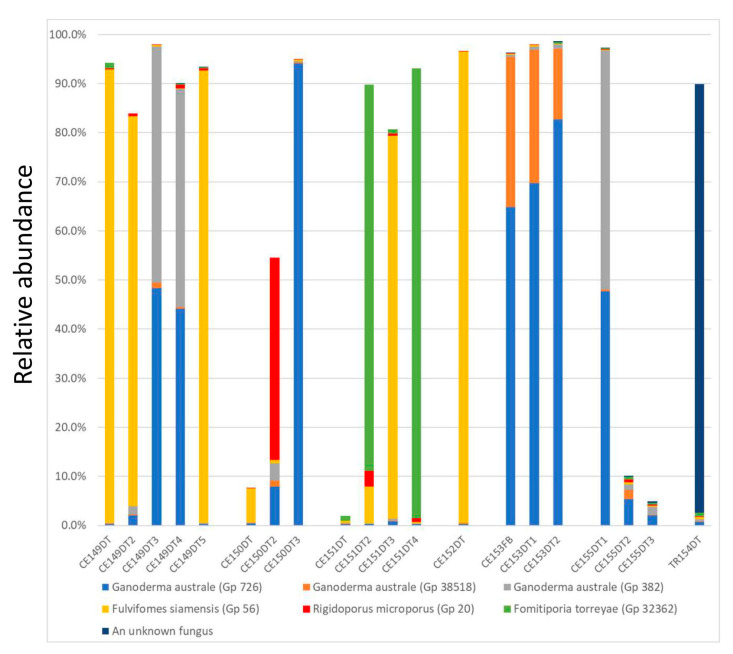
Fungi genetic heterogeneity in Casuarina’s diseased tissues. All the seven trees were located near each other at the West Coast Park, Singapore. A fruiting body and multiple decayed wood samples were subjected to a metagenomic analysis, and the relative abundances of selected OTUs are presented in the bar chart (as percentages of the total sequence reads for the sample). GenBank accession numbers: OQ572592 for Gp726/OTU_47_Apr20; OQ572594 for Gp38518/OTU_137_Apr20; OQ572593 for Gp382/OTU_120_Aug20; OQ572588 for Gp56/OTU_519_Apr20; OQ572639 for Gp20/OTU_2557_Apr20; OQ572584 for Gp32362/ASV3_Mar22.

Figure 6 showcases the genetic heterogeneity in the diseased tissues collected from multiple Casuarina trees at one site.

Among the five diseased tissues collected from CE149, three of them were dominated by *Fulvifomes siamensis*, while the other two samples were dominated by two OTUs of *Ganoderma australe* (Gp726 and Gp382) with a minor presence of a third *G. australe* OTU (Gp38518). One *G. australe* (Gp727) OTU was the single dominant presence for CE150DT3, and also in the three diseased tissue samples from CE153, together with the third *Ganoderma australe* OTU (Gp38518). *Rigidoporus microporus,* on the other hand, had a significant presence in one diseased tissue from CE150 (CE150DT2). *Fomitiporia torreyae* was the dominant presence in two diseased tissues underneath the crust fruiting bodies on the tree branch, and one of the two samples (CE151DT2) also had a minor presence of *Fulvifomes siamensis*, which was the dominant pathogen for another diseased tissue from the same tree (CE151DT3); it was also the dominant presence for the diseased tissue collected from its neighbor (CE152DT). Multiple fruiting bodies collected from these trees were barcoded as *Fulvifomes siamensis*.

A decayed wood sample was also collected from a Trumpet tree (TR154) at the same location from its trunk at 1 m above the ground. The dominant fungus in the decayed wood was unknown, a possible new fungus specific to the Trumpet tree.

### 3.7. Genetic Heterogeneity in Fruiting Bodies

Direct barcoding of the fruiting bodies sometimes did not yield clear results. A visual inspection of sequence chromatograms often reveals poor quality sequencing regions with overlapping peaks, suggesting genetic heterogeneity. A metagenomic analysis of these fruiting bodies confirmed the genetic heterogeneity within a fruiting body. Examples include:

- *Fulvifomes siamensis:* A fruiting body from Rain Tree (R28FB3) contained two OTUs of *Fulvifomes siamensis* (OQ572589/OTU_1315_Deca20 and OQ572586/OTU_7_Deca20) and two *Trechispora* sp. OTUs (OQ589717/OTU_9_Deca20 and OQ589718/OTU_552_Deca20) with relative abundances of 36%, 13%, 22% and 27%, respectively.
- *Phellinus noxius*: Three OTUs were found in one fruiting body (L129FB) with relative abundances of 34%, 15% and 50%.
- *Fomes meliae*: A fruiting body collected from an Angsana tree (A127FB) contained two OTUs of this fungus with 46% and 53% relative abundance.

**Figure 7:**
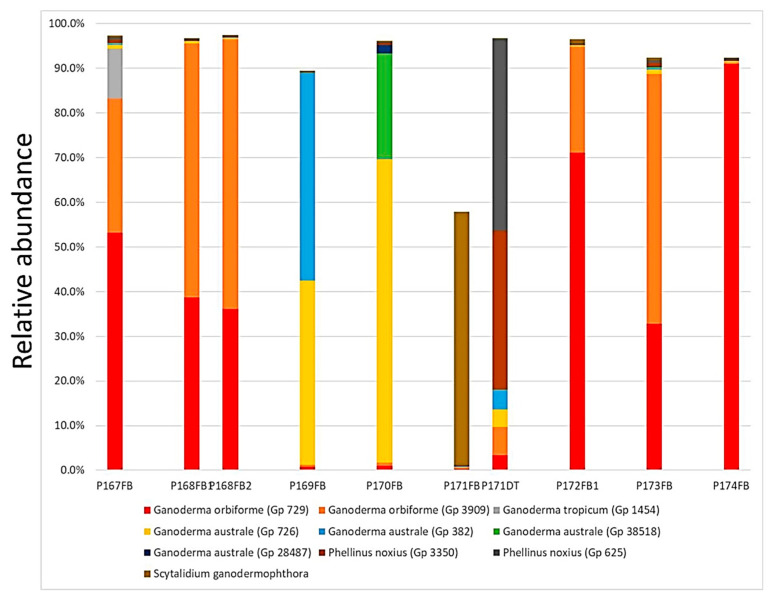
Genetic heterogeneity in *Ganoderma* fruiting bodies. Multiple fruiting body samples were subjected to a metagenomics analysis and the relative abundance of OTUs is represented in the bar chart as a percentage of the total sequence reads for the sample. GenBank accession numbers: OQ572598 for Gp729/OTU_360_Apr20; OQ572601 for Gp1454/OTU_1139_Apr20; OQ572592 for Gp726/OTU_47_Apr20; OQ572599 for Gp3909/OTU_1796_Jun21; OQ572593 for Gp382/OTU_120_Aug20; OQ572594 for Gp38518/OTU_137_Apr20; OQ572590 for Gp28487/ASV525_Jun22; OQ572614 for Gp3350:/OTU_17_Apr21; OQ572621 for Gp625/OTU_846_Aug20; *S. ganodermophthora*: OQ572648.

Genetic heterogeneity within the fruiting body is mostly prominent in the *Ganoderma* species. Figure 7 details the genetic heterogeneity in many *Ganoderma* fruiting bodies collected from one location (Clementi Ave 5). Except for P174FB, which was dominated by a single *G. orbiforme* (90.9% relative abundance) OTU, all other fruiting bodies were genetically heterogenous. P167FB was a mixture of two OTUs of *G. orbiforme* and one strain of *G. tropicum* (with relative abundances of 53.1%, 30.1% and 11.2%, respectively), while two fruiting bodies from a Foxtail Palm (P168FB1/FB2), one fruiting body from another Foxtail Palm (P173FB) and one fruiting body from a Yellow Cane Palm (P172FB) were mixtures of two *G. orbiforme* OTUs. Among the five *G. australe* OTUs identified, two of them were the major presence in the two fruiting bodies collected from two palms (P169FB, P170FB). A noticeable exception was P171FB, which was dominated by *Scytalidium ganodermophthora*, a parasitic ascomycota for *Ganoderma*. The P171 decayed stem was dominated by two *Phellinus noxius* OTUs (42.9% and 35.6% relative abundance), also with the presence of two OTUs of *G. orbiforme* and two OTUs of *G. australe* (with relative abundances of 3.4%, 6.4%, 3.9% and 4.3%, respectively).

### 3.8. Inter-Strain Heterogeneity vs. Intra-Strain Heterogeneity for Ganoderma Species

A metagenomic analysis provides the unique opportunity to separate different genotypes apart in the same sample. There are two possible reasons for the presence of multiple genotypes of one species within the same fruiting body: (1) inter-strain heterogeneity among strains in a mosaic fruiting body; (2) intra-strain heterogeneity due to variation among rDNA copies within the same strain. One strategy was used to differentiate the two possibilities: if one strain contains multiple genotypes (intra-strain heterogeneity), these genotypes will co-exist in all samples containing this strain. They would exhibit the same/similar sequence read abundance in all the samples. With this simple strategy, few intra-strain heterogeneity cases could be identified, but most genotypes do not fit into this criterion, and hence belong to different strains (inter-strain heterogeneity).

**Figure 8:**
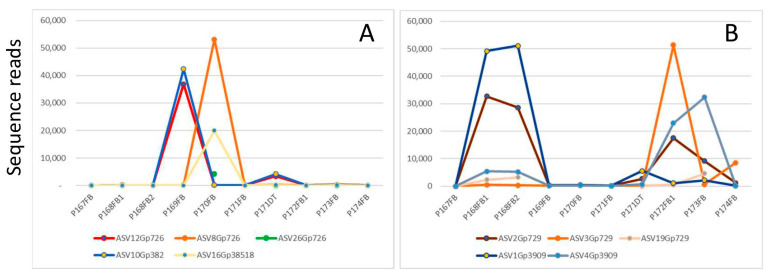
Intra-strain heterogeneity and inter-strain heterogeneity for *Ganoderma* species. Nine *Ganoderma* fruiting bodies and one decayed palm stem were collected from the same location (Clementi Avenue 5) and subjected to metagenomic analysis. Abundance for the *Ganoderma* genotypes is represented by their sequence reads in each sample. (**A**) *Ganoderma australe*: OQ566790/ASV12Gp726, OQ566788/ASV26Gp726, OQ566792/ASV8Gp726 are three genotypes of the Main OTU Gp726, and OQ566791/ASV10Gp382 and OQ566789/ASV16Gp38518 belong to the Main OTUs Gp382 and Gp38518, respectively. (**B**) *Ganoderma orbiforme*: OQ566787/ASV1Gp3909 and OQ566784/ASV4Gp3909 are two genotypes of the Main OTU Gp3909, and OQ566786/ASV2Gp729, OQ566785/ASV3Gp729 and OQ566783/ASV19Gp729 are three genotypes of the Main OTU Gp729.

Figure 8 compares the abundance of five genotypes of *Ganoderma australe* (Figure 8A) and five genotypes of *Ganoderma orbiforme* (Figure 8B) in nine fruiting bodies and one decayed palm stem collected from the same location. ASV12Grp726 and ASV10Gp382 were present in tandem with each other in all the samples, especially in P169FB and P171DT. Such a perfect co-existence of genotypes indicates intra-strain heterogeneity, and this finding is consistent with the report of intra-strain ITS heterogeneity in the four *Ganoderma* species studied [29].

The other three genotypes for *G. australe* and five genotypes for *G. orbiforme* had distinctive distribution profiles, suggesting that they belong to different strains and their co-existence is the result of a mixture of different strains in a single fruiting body (mosaic fruiting body). Our findings are consistent with the findings of fruiting body formation involving genetically heterologous mycelia for *Lentinula edodes* and *Armillaria gallica* [30,31].

Another *Phellinus noxius* fruiting body (L129FB) was identified with three distinct OTUs (OTU_179_Oct21, OTU_458_Oct21, OTU_12_Oct21). Since this tri-genotype combination was not found in any other sample, we suggest that this fruiting body was a mosaic of multiple strains, which might have facilitated the formation of the fruiting body. Inter-strain heterogeneity was also identified in diseased tissues infected by *Phellinus noxius*, *Fulvifomes siamensis* and *Ganoderma orbiforme*.

### 3.9. In Vitro Wood Decay Studies

In vitro wood decay studies were conducted by inoculating sterile dry wood blocks with pure isolates of fungi (*Fulvifomes* siamensis: OQ618213; *Phellinus noxius*: OQ558864; *Rigidoporus microporus*: OQ558869). After incubation for a period of 12 weeks, wood weight loss was assessed as the indication of wood decay caused by the inoculated fungus. In total, 20 wood blocks were used for each treatment and another 20 wood blocks were treated the same way but without fungal inoculation to serve as the reference group.

**Figure 9:**
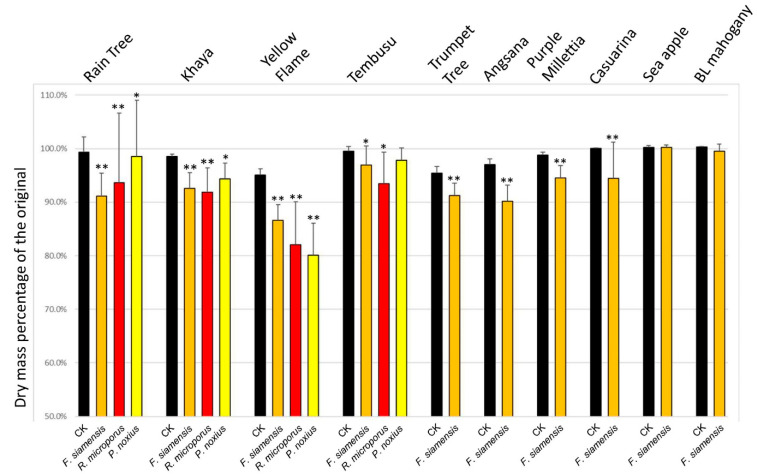
In vitro wood decay studies. Double autoclaved dry wood blocks (30 × 10 × 8 mm) were incubated with PDA Petri dishes (90 mm diameter) fully colonized by the respective fungi. Dishes were sealed and incubated at 28 °C for 12 weeks. After removing the fungus and drying, the wood blocks were weighed and presented as percentages of their original weights (n = 20 for each inoculation). CK: reference group, wood blocks going through the same procedure but without fungus inoculation (n = 20). ** Very significant difference between a treatment group and its reference group (CK) by Student’s *t*-test (*p* < 0.01); * Significant difference from CK by Student’s *t*-test (0.01 < *p* < 0.05).

The results are summarized in Figure 9.

For *Fulvifomes siamensis*, the in vitro pathogenicity studies proved its capability of breaking down the wood of several different urban trees in Singapore. Seven of the ten tree species studied had significant wood mass loss (*p* < 0.01) after incubation with its pure isolate for 12 weeks, with that of the Yellow Flame reaching 20% weight loss, and with the wood mass loss of Tembusu wood being significant at the 0.01 < *p* < 0.05 level. On the other hand, there were no significant wood mass losses for the Sea Apple and Broad Leaf Mahogany trees.

*Rigidoporus microporus* similarly led to significant wood weight loss in Rain Tree, Khaya and Yellow Flame wood (*p* < 0.01) but was only significant at the *p* < 0.05 level with respect to the Tembusu wood.

*Phellinus noxius* performed more poorly than the above two. While causing significant wood loss for Yellow Flame wood (*p* < 0.01), this was only significant at the 0.01 < *p* < 0.05 level for Rain Tree and Khaya wood, and not significant for the Tembusu wood. The conclusion of weak pathogenicity for the Khaya tree seems not fully agreeable with our finding of multiple Khaya trees infected by *Phellinus noxius*. This suggests that it requires a longer time to infect a Khaya tree due to its high wood density. Another possible reason is that the strain we used in this study might be less pathogenic.

*Phellinus noxius* also did not result in significant wood mass loss with the Tembusu wood, which is a result consistent with the absence of this fungus in the Tembusu diseased tissues analyzed.

## 4. Discussion

The *Polyporales* are an order of fungi within the phylum *Basidiomycota*, commonly known as bracket fungi or polypores. These fungi are characterized by the presence of pores or small holes on the underside of the fruiting body (basidiocarp) through which spores are released. Polyporales are typically wood-decaying fungi and play important ecological roles in the decomposition and nutrient cycling of forest ecosystems. Our project has shortlisted nine Polyporales as potential problems for the urban trees of Singapore, with their presence in the diseased tissues, and also in the surrounding soil and/or fruiting bodies. *Rigidoporus microporus* infects mostly the Rain Tree and Casuarina. *Ganoderma orbiforme* is a possible problem for ornamental palms, while *Ganoderma australe* infects both palms and Casuarina trees. There are fewer cases of infection by the other six Polyporales, and all in Angsana trees.

The order *Hymenochaetales* of white rot fungi has some of the most aggressive wood decayers causing tree deaths around the world. Our project has identified four members that are potentially pathogenic to different urban trees in Singapore: *Fulvifomes siamensis*, *Phellinus noxius*, *Fomitiporia bannaensis* and *Fomes meliae*. *Fulvifomes siamensis* has the widest host range among all the shortlisted fungi. Its potential pathogenicity to a wide range of urban trees was further supported by the in vitro wood decay study.

The consistent presence of *Phellinus noxius* as mycelia in bark and decayed sap wood strongly suggests its pathogenicity to the Khaya tree, a common roadside tree. Its presence in soil samples for the Khaya tree provides further support.

A survey of root and butt rot fungi in the USA Pacific Islands identified *Fomitiporia bannaensis* fruiting bodies on Casuarina trees [32]. On the other hand, it has been detected in dead *Camellia oleifera*, *Castanospsis* and *Rhododendron* in China [33]. Its status as either a plant pathogen, a wood decay fungus or somewhere in between will have to be determined through Koch’s postulate.

For *Fomes meliae,* it has only been reported as a disease in temperate trees; therefore, its pathogenicity towards Angsana will have to be determined through Koch’s postulate experiments.

*Serpula similis* is a brown rot fungus first identified in the diseased tissues of Angsana. *Serpula similis* is probably a paleotropical species, frequently collected on bamboo including Calcutta bamboo (*Dendrocalamus strictus*), both in the wild and in buildings, but also on other wood such as Leucaena glauca in central India [34,35]. In comparison, a related species, *Serpula lacrymans*, has been regarded as the most dangerous wood decay fungus in Europe, accounting for 61.5% of wood structure damage as revealed by a survey in Styria, Austria [36]. It is, however, mostly found inside the built environment in Europe, with no report of its presence in the Tropics. As such, Koch’s postulate needs to be carried out to determine *S. similis*’ relationship with its host, especially when there are no reports of *S. similis* as a plant pathogen.

In addition to the shortlisted fungi, the project also identified several other fungi that are less likely to be pathogenic to Singapore urban trees but have their own interests, such as the following:

- *Scytalidium ganodermophthora* detected in one *Ganoderma*-like fruiting body was suggested to have hyperparasitized the *Ganoderma* fruiting body. It holds some potential for the biocontrol of the *Ganoderma* species that is known to be difficult to control in field conditions.
- While *Perenniporia fraxinea* (*Vanderbylia fraxinea*) can cause infections in a large number of trees, it has not been reported as such in tropical countries [37], and Koch’s postulate needs to be applied to determine the actual pathogenic and/or saprophytic relationship with the Rain Tree.
- *Phellinus pachyphloeus* was first detected in Singapore.

A metagenomic analysis revealed the presence of multiple fungi and their relative abundance in most diseased tissue samples, and this confirms the speculation of fungal communities in wood decay [20]. One example is the Yellow Flame tree (YF37) with multiple *Fulvifomes siamensis* fruiting bodies, but this fungus had little presence in the decay wood samples, which were dominated by *Fomitiporia bannaensis* and *Gloiothele lactescens.* This might suggest a primary *Fulvifomes* infection that is followed by successive secondary infections by the other fungi.

The results from multiple infected Casuarina trees at one location (Figure 6) highlight the complexity of pathogenesis by wood decay fungi. Decayed wood tissues in different locations of the same tree dominated by different fungal species suggest their infections occurred independently. The co-existence of multiple fungal species and multiple strains of the same species may suggest fungal collaboration in wood decay.

Another key finding of our project is the presence of genetic heterogeneity in fungal fruiting bodies that is mostly prevalent in *Ganoderma* species. *Ganoderma* fruiting bodies are often mosaics of more than one species and of multiple strains of the same species (Figure 7). It was further proven that both intra-strain heterogeneity and inter-strain heterogeneity were present in the *Ganoderma australe* fruiting bodies (Figure 8), and inter-strain heterogeneity prevailed.

Genetic heterogeneity in fungi can have important implications for a variety of processes, including adaptation, speciation and pathogenicity. For example, genetic heterogeneity can allow fungi to adapt to changing environmental conditions or to evade host immune defenses. In pathogenic fungi, genetic heterogeneity can contribute to the emergence of drug-resistant strains, making it more difficult to treat infections. Inter-genetic heterogeneity (mosaic fruiting body) was identified in Armillaria gallica [31] and was suggested to be a contributing factor to the longevity and capacity for continuous growth. A follow-up study of single hyphal filament clones confirmed this inter-strain heterogeneity and uncovered the intra-strain heterogeneity as well [38]. These two types of genetic heterogeneity were suggested to give fungal individuals the potential to evolve within a single generation in response to environmental variation over time and space.

Our in vitro wood decay studies generally support the metagenomic and fruiting body barcoding results. Wood loss after fungal inoculation is more obvious for the tree species with lighter wood such as the Rain Tree and Yellow Flame. Wood loss is less obvious for high-density hard wood species such as Sea Apple, Khaya tree and Broad Leaf Mahogany. This suggests that these trees may need more time for fungal hyphae to penetrate; therefore, wood decay may not be obvious after 12 weeks of incubation. Another possible reason is the virulence variation among strains. The isolates we used might not be the most virulent. This suggestion is supported by a recent report of five strains of *R. microporus* isolated from diseased rubber trees. They all had 99% sequence identity to *R. microporus* isolate SEG but showed variations in virulence when tested on rubber tree seedlings [2]. Another possibility is the need for other factors or conditions for infection to start.

Our findings also provide some explanations for the difficulty of early diagnosis of pathogenic fungi. *Rigidoporus microporus* has been detected in a number of diseased Rain Trees, but only a small number of them had visible growth of *Rigidoporus microporus* fruiting bodies. It is suggested that the formation of fruiting bodies is not the only indication of root wood decay, as some trees had decayed root, but no fruiting bodies could be found. This makes early diagnosis based on the presence of a fruiting body irrelevant. A similar situation happened with the *Phellinus noxius* infection of Khaya trees. In the period of 2020–2022, more than 20 diseased Khaya trees were found infected by *Phellinus noxius* but none of them exhibited any fruiting body, not even in those severely damaged by this fungus. It is suggested that fruiting body formation is not a good indicator for incipient root/wood decay, and alternative targets such as the presence of pathogenic fungi in soil should be explored for the purpose of early diagnosis.

## 5. Conclusions

As a summary of the research, this first-of-its-kind comprehensive survey of fungi was performed for diseased urban trees in Singapore, a tropical country well-recognized for its urban greenery. Through the combinational use of a metagenomic analysis of >200 soil samples and diseased tissues with fruiting body barcoding, comprehensive knowledge has been accumulated on such fungi for tropical urban trees. The potential pathogenicity of some of these fungi was further boosted by the in vitro wood decay study, in which fungal isolates caused the significant dry mass loss of inoculated wood blocks. The tree species surveyed are common species grown in parks and along roadsides in Singapore, other Southeast Asia countries and many other tropical and subtropical cities. Our findings will serve as a reference for tropical urban tree managers. The findings of genetic heterogeneity in diseased tissues further support the possible complexities of multiple fungal infections. The intra-strain and inter-strain genetic heterogeneity in fruiting bodies suggests possible ecological advantages from the mixture of genotypes, which warrant further investigation. Finally, the findings lay the foundation for the performing of early diagnosis and also allow the precise design of mitigation strategies. The significant funga diversity uncovered by this project will become a valuable resource for other research projects on tropical fungi.

## Figures and Tables

**Table 1:** Summary tabulation of the soil, tissue and fruiting body samples taken from the respective trees.

Species	Common Names	Tree Code	Number of Trees	Soil Samples	Tissue Samples	Fruiting Bodies	Total for Each Species
*Samanea saman*	Rain Tree	R	36	38	28	23	89
*Pterocarpus indicus*	Angsana	A	18	11	24	22	57
*Khaya Senegalensis*	Khaya	KS	19	13	27	7	47
*Casuarina equisetifolia*	Casuarina	CE	12	7	20	16	43
*Peltophorum pterocarpum*	Yellow Flame	YF	15	13	8	21	42
*Syzygium grande*	Sea Apple	SG	7	2	3	9	14
*Swietenia macrophylla*	Broad Leaf Mahogany	SM	5	4	5	3	12
*Cyrtophyllum fragrans*	Tembusu	CF	2	2	2	0	4
*Tabebuia rosea*	Trumpet Tree	TR	3	2	1	0	3
*Callerya atropurpurea*	Purple Millettia	PM	2	0	5	0	5
*Wodyetia bifurcata*	Foxtail Palm	P	4	0	0	5	5
*Dypsis lutescens*	Yellow Cane Palm	P	4	0	1	5	6
*Sabal palmetto*	Cabbage Palm	P	1	0	0	1	1
*Cyrtostachys renda*	Red Sealing Wax Palm	P	6	6	0	0	6
	Unidentified Log	L	6	0	1	9	10
	Primary Forest Reference		3	3			3
	Total		143	101	125	121	347

**Table 2:** Summary of the funga diversity in Singapore.

Phylum	OTUs	*Ascomycota* Classes	OTUs	*Agaricomycetes* Orders	OTUs
*Ascomycota*	6826	*Archaeorhizomycetes*	155	*Polyporales*	237
*Basidiomycota*	2426	*Dothideomycetes*	1237	*Agaricales*	688
*Chytridiomycota*	224	*Leotiomycetes*	484	*Amylocorticiales*	1
*Zygomycota*	335	*Lecanoromycetes*	67	*Atheliales*	11
*Glomeromycota*	335	*Saccharomycetes*	157	*Auriculariales*	24
*Rozellomycota*	213	*Sordariomycetes*	2556	*Boletales*	192
*Mortierellomycota*	152	*Coniocybomycetes*	1	*Cantharellales*	113
*Mucoromycota*	84	*Arthoniomycetes*	3	*Corticiales*	13
*Kickxellomycota*	39	*Ascomycetes*	0	*Geastrales*	42
*Zoopagomycota*	13	*Eurotiomycetes*	1198	*Gloeophyllales*	1
*Calcarisporiellomycota*	11	*Pezizomycetes*	141	*Gomphales*	6
*Olpidiomycota*	10	*Geoglossomycetes*	3	*Hymenochaetales*	116
*Neocallimastigomycota*	6	*Umbelopsidomycetes*	20	*Hysterangiales*	4
*Aphelidiomycota*	3	*Laboulbeniomycetes*	5	*Phallales*	13
*Basidiobolomycota*	2	*Orbiliomycetes*	107	*Russulales*	81
*Entorrhizomycota*	1	*Xylonomycetes*	4	*Sebacinales*	73
*Monoblepharomycota*	1	***Basidiomycota* classes**	OTUs	*Thelephorales*	93
*Blastocladiomycota*	7	*Agaricomycetes*	1983		
*Olpidiomycota*	10	*Agaricostilbomycetes*	9	***Polyporales* families**	**OTUs**
Others	77	*Atractiellomycetes*	8	*Cerrenaceae*	1
**Total**	10775	*Classiculomycetes*	1	*Fomitopsidaceae*	11
		*Cystobasidiomycetes*	9	*Ganodermataceae*	49
		Dacrymycetes	3	*Incertae_sedis_Polyporales*	1
		*Microbotryomycetes*	46	*Irpicaceae*	8
		*Tremellomycetes*	191	*Meripilaceae*	19
		*Exobasidiomycetes*	7	*Meruliaceae*	13
		*Geminibasidiomycetes*	2	*Phanerochaetaceae*	16
		*Wallemiomycetes*	11	*Podoscyphaceae*	4
		*Malasseziomycetes*	15	*Polyporaceae*	34
		*Moniliellomycetes*	1	*Polyporales_fam_Incertae_sedis*	7
		*Ustilaginomycetes*	28	*Steccherinaceae*	3
		*Spiculogloeomycetes*	1	unidentified_Polyporales_sp	2
		*Pucciniomycetes*	9	*Xenasmataceae*	6
				***Hymenochaetales***	**OTUs**
				*Hymenochaetaceae*	
				*Fulvifomes*	16
				*Phellinus*	29
				*Pyrrhoderma*	10

**Table 3:** The short list of wood decay fungi for Singapore urban trees. The presence of each fungus in the soil, as the fruiting body and inside diseased tissues is represented by tree codes followed by the number of trees and total number of samples in brackets. GenBank accession numbers for the main OTUs are also included.

Pathogenic Fungi	Type	Main OTUs	Presence in Soil (Trees/ Samples)	As Fruiting Bodies (Trees/Samples)	Inside Diseased Tissues (Trees/Samples)	GenBank Accession Number/Main OTU ID
*Rigidoporus microporus*	*Polyporales*, white rot	13	R(16/19), CE(7), YF(1)	R(5), P(1/2)	R(6/15), CE(3), KS(1), YF(1)	OQ572639/OTU_2557_Apr20 OQ572631/OTU_5_Jan21 OQ572638/OTU_1930_Decb20 OQ572635/OTU_127_Decb20 OQ572637/OTU_1592_Decb20 OQ572628/OTU3_AprGW21 OQ572629/OTU_2_Aug20 OQ572632/OTU_52_Mara21 OQ572640/OTU_5758_Deca20 OQ572630/OTU_3_Deca20 OQ572634/OTU_78_Mara21 OQ572633/OTU_74_Mara21 OQ572636/OTU_1379_Oct21
*Meripilus giganteus*	*Polyporales*, white rot	1		A(1)	A(1)	OQ572606/OTU_1147_Dec21
*Ganoderma orbiforme*	*Polyporales*, white rot	3	P(5)	P(5/6),	P(1)	OQ572599/OTU_1796_Jun21, OQ572598/OTU_360_Apr20 OQ572597/OTU_187_Jul20
*Ganoderma australe*	*Polyporales*, white rot	7	P(3), CE(3), R(5), A(1)	P(3), CE(1), R(1)	P(1), CE(5/9),	OQ572593/OTU_120_Aug20 OQ572592/OTU_47_Apr20 OQ572590/ASV525_Jun22 OQ572596/OTU_5865_Jul20 OQ572591/OTU_10_Oct21 OQ572594/OTU_137_Apr20 OQ572595/OTU_481_Oct21
*Tomophagus colossus*	*Polyporales*, white rot	1		A(2)	A(1)	OQ572668/OTU_853_Jun21
*Fomes meliae*	*Polyporales*, brown rot	5		A(1)	A(2)	OQ572579/OTU_58_Dec21 OQ572577/OTU_9_Oct21 OQ572580/OTU_178_Oct21 OQ572576/ASV14_May22 OQ572578/OTU_51_Dec21
*Perenniporia tephropora*	*Polyporales*, crust fungi for white rot	1	SM(1)	A(1)	A(1)	OQ572608/OTU_1786_Aug20
*Pseudofibroporia citrinella*	*Polyporales*, white rot	2		A(1)	YF(1/2)	OQ572626/OTU_45_Jan21 OQ572627/OTU_46_Jan21
*Flavodon flavus*	*Polyporales*, white rot	1		A(1)	A(1/2)	OQ572575/ASV2_May22
*Fulvifomes siamensis*	*Hymenochaetales*, white rot	5	R(9/10), CE(5), YF(4), KS(5/6), SG(2), P(6), SM(2)	R(10/16), CE(8/13), YF(12/18), KS(4), SG(6/8), A(1), SM(3)	R(3/4), CE(4), YF(1/2), KS(3/5), SG(2/3), A(3/5), SM(2), CF(1)	OQ572588/OTU_519_Apr20 OQ572585/OTU5_AprGW21 OQ572587/OTU_12_Marb21 OQ572589/OTU_1315_Deca20 OQ572586/OTU_7_Deca20
*Phellinus noxius*	*Hymenochaetales*, white rot	16	KS(3), R(2), CF(1), SM(2), CE(1)	YF(1/2)	KS(9/18), R(1), CF(1), P(1), CE(1), SM(1)	OQ572621/OTU_846_Aug20 OQ572614/OTU_17_Apr21 OQ572610/OTU74_AprGW21 OQ572609/ASV198_Jun22 OQ572619/OTU_319_Mara21 OQ572618/OTU_300_Marb21 OQ572613/OTU_12_Oct21 OQ572615/OTU_30_Marb21 OQ572611/OTU_2_Marb21 OQ572622/OTU_2335_Dec21 OQ572624/OTU_3632_Jun21 OQ572612/OTU_6_Oct21 OQ572616/OTU_130_Jun21 OQ572620/OTU_458_Oct21 OQ572617/OTU_179_Oct21 OQ572623/OTU_2936_Dec21
*Fomitiporia bannaensis*	*Hymenochaetales*, white rot	3	SM(1)		YF(1), KS(3)	OQ572581/OTU_16_Apr21 OQ572583/OTU_2535_Decb20 OQ572582/OTU_1321_Oct21
*Fomitiporia torreyae*	*Hymenochaetales*, white rot	1	CE(1)	CE(1)	CE (1/2)	OQ572584/ASV3_Mar22
*Serpula similis*	*Boletales*, brown rot	8	A(1/3), R(1)	A(1)	A(2/5), KS(1)	OQ572662/OTU_26_Apr21 OQ572663/OTU_47_Mara21 OQ566782/OTU_48_Mara21 OQ572664/OTU_63_Mara21 OQ572666/OTU_926_Aug20 OQ572665/OTU_526_Decc20 OQ572661/OTU1_AprGW21 OQ572667/OTU_1321_Decc20
*Scytalidium lignicola*	*Ascomycota*	5			A(3/6), PM(2), CE(1)	OQ572652/OTU_1526_Jul20 OQ572653/OTU_1561_Jul20 OQ572651/OTU_296_Oct21 OQ572650/ASV22_May22 OQ572660/OTU_11_Decc20
*Scytalidium circinatum*	*Ascomycota*	10	A(1), CE(3), KS(1), R(1), YF(1)		A(3/4)	OQ572641/OTU685_AprGW21 OQ572654/OTU2_AprGW21 OQ572655/OTU_5_Decc20 OQ572656/OTU_21_Dec21 OQ572657/OTU_41_Mara21 OQ572658/OTU_148_Dec21 OQ572659/OTU_284_May21 OQ572644/OTU_294_Decb20 OQ572642/OTU_12_Dec21 OQ572643/OTU_82_Decb20
*Scytalidium cuboideum*	*Ascomycota*	3		R(1)	A(1) R(1)	OQ572647/OTU_516_Deca20 OQ572645/OTU_13_Aug20 OQ572646/OTU_20_Deca20

## Data Availability

More data supporting the reported results can be found in the Supplementary Materials detailed.

## Supplementary Materials

The following supporting information can be downloaded at: https://www.mdpi.com/article/10.3390/jof9040460/s1, Table S1: Sample collection details; Table S2: A database of funga diversity for Singapore urban trees.
